# Correction: Bayesian parameter estimation for dynamical models in systems biology

**DOI:** 10.1371/journal.pcbi.1011041

**Published:** 2023-04-05

**Authors:** Nathaniel J. Linden, Boris Kramer, Padmini Rangamani

In section 1.6 Constrained interval unscented Kalman filter Markov chain Monte Carlo (CIUKF-MCMC) of the Materials and Methods section, there is an error in Theorem 1. Specifically, in several of the equations in the theorem, some of the indices on x and y in the exponents are incorrect. Please find the correct theorem below;

**Theorem 1 (Marginal likelihood (Theorem 1 of [26] and 12.1 of [67]))** *Let y_k_ denote the set of all observations up to time t_k_ as defined in Section 1.2*. *Let the initial condition be uncertain with distribution p* (**x**_0_|***θ***). *Then the marginal likelihood is defined recursively in three stages*:

*for k =* 1,2,…

1. *Predict the new state from previous data*
$$p\;({\text{x}}_{k+1}❘\theta ,y_{k})=\int_{{\mathbb{R}}_{\geq 0}^{d}}p\;({\text{x}}_{k}❘\theta ,y_{k})\frac{\exp\;(-\frac{1}{2}||{\text{x}}_{k+1}-\psi ({\text{x}}_{k},\theta_{f})|{|}_{\sum }^{2})}{{\left(2\pi \right)}^{\frac{d}{2}}|\sum (\theta_{\sum }){|}^{\frac{1}{2}}}{\text{d}\text{x}}_{k}\text{,}$$
2. *update the prediction with the current data*
$$p\;({\text{x}}_{k+1}❘\theta ,y_{k+1})=p\;({\text{x}}_{k+1}❘\theta ,y_{k})\frac{\exp\;(-\frac{1}{2}❘❘{\text{y}}_{k+1}-{\text{Hx}}_{k+1}❘{❘}_{\Gamma }^{2})}{{\left(2\pi \right)}^{\frac{m}{2}}❘\Gamma (\theta_{\Gamma }){❘}^{\frac{1}{2}}}\text{,}$$
3. *and marginalize out uncertainty in the states*
$$L_{k+1}(\theta ❘y_{k+1})=\int_{{\mathbb{R}}_{\geq 0}^{d}}\;p\;({\text{x}}_{k+1}❘\theta ,y_{k})\frac{\exp\;(-\frac{1}{2}❘❘{\text{y}}_{k+1}-{\text{Hx}}_{k+1}❘{❘}_{\Gamma }^{2})}{{\left(2\pi \right)}^{\frac{m}{2}}❘\Gamma (\theta_{\Gamma }){❘}^{\frac{1}{2}}}{\text{d}\text{x}}_{k+1}\text{.}$$
