# Supplementary figures and images for: Bayesian parameter estimation for dynamical models in systems biology
Source: PLoS Comput Biol. 2022 Oct 21;18(10):e1010651. doi: 10.1371/journal.pcbi.1010651 (PMC9629650; doi:10.1371/journal.pcbi.1010651)

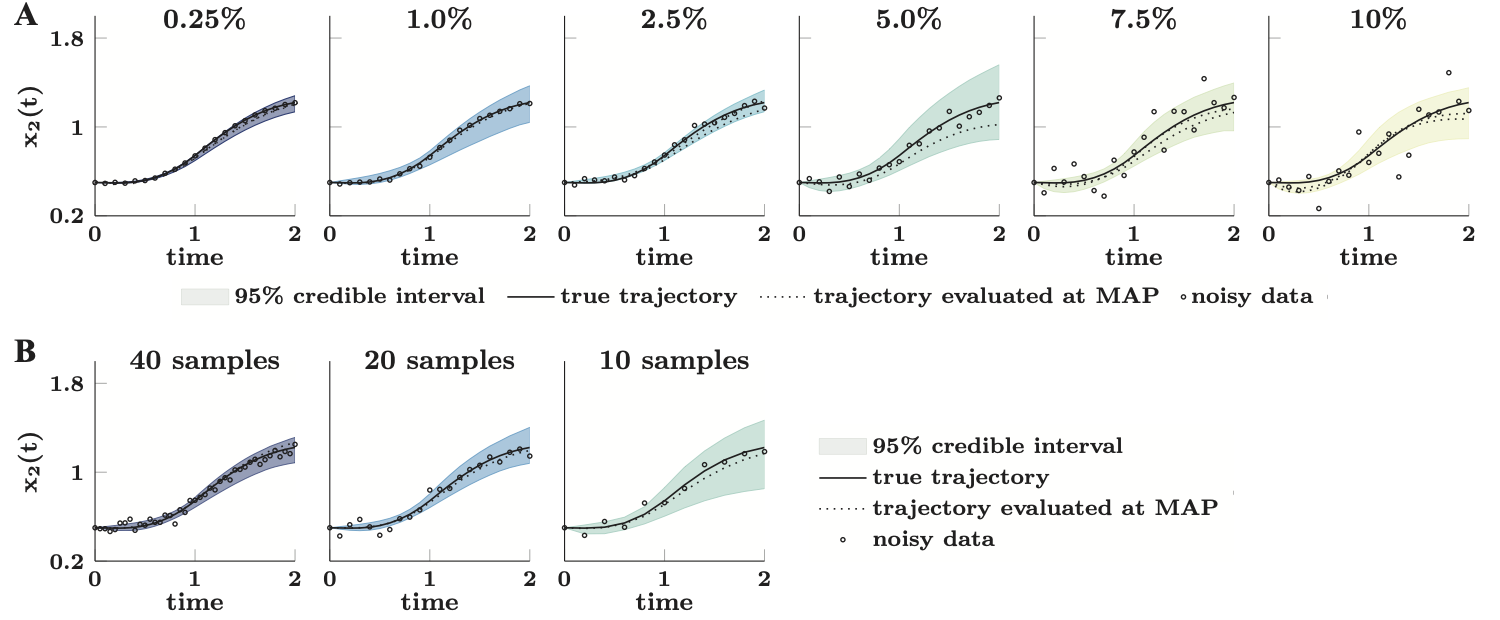

Supplement: S1 Fig — (A) corresponds to the measurement noise experiment, Fig 3C. We observe that increasing the data noise level increased the uncertainty in the predicted dynamics. We control the noise level by setting the noise covariances to the specified percentage of the standard deviation of each state variable. The dashed black vertical lines indicate each parameter’s nominal (true) value. (B) corresponds to the data sparsity experiment, Fig 3E. We observe that decreasing the number of experimental samples supplied for estimation increased estimation uncertainty. The noise level in the data was fixed to the 2.5% level shown above. (TIF) [file pcbi.1010651.s001.tif]

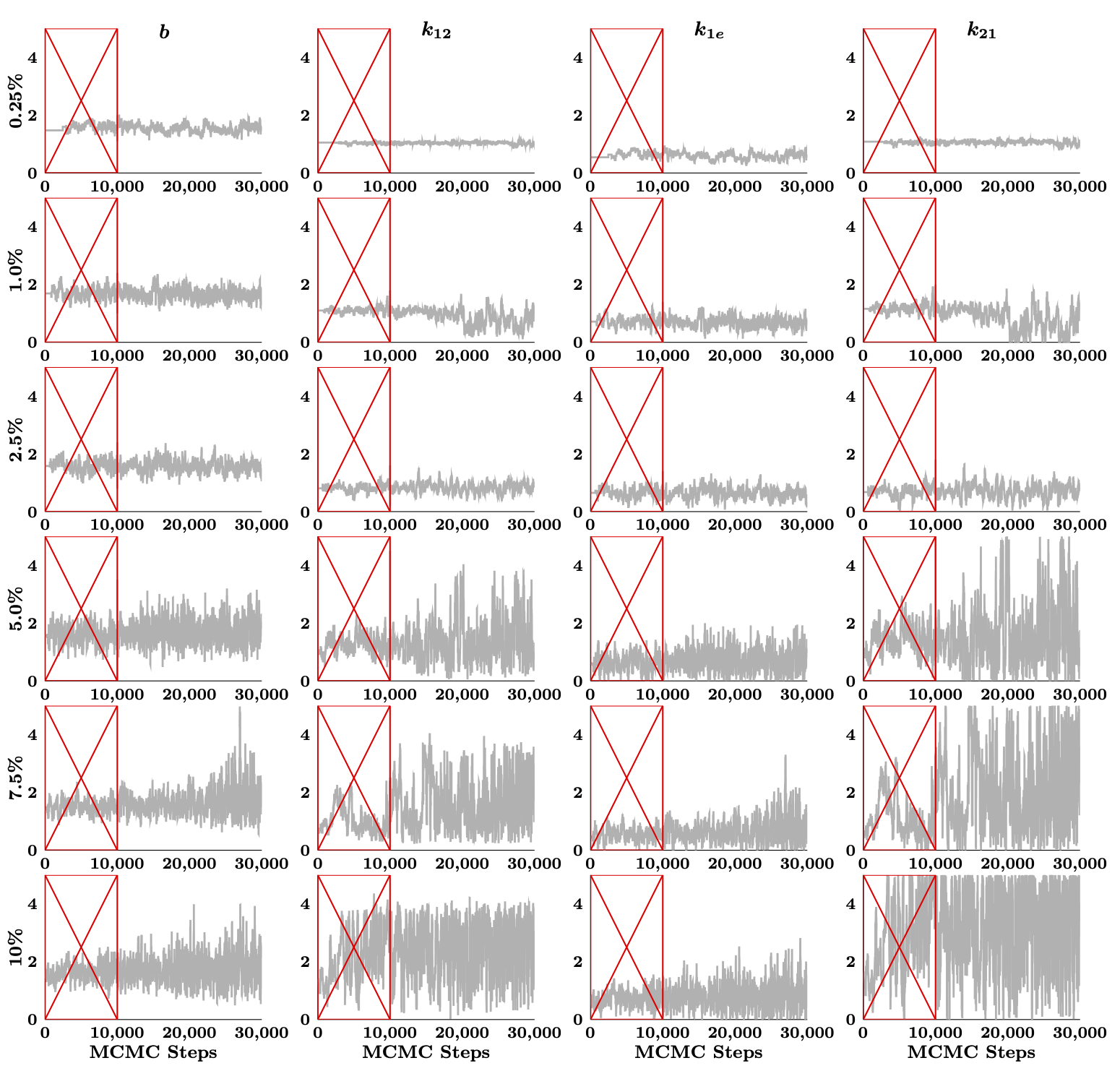

Supplement: S2 Fig — Each row corresponds to a noise level; noise increases down the figure. The red boxes indicate samples discarded as burn-in. (TIF) [file pcbi.1010651.s002.tif]

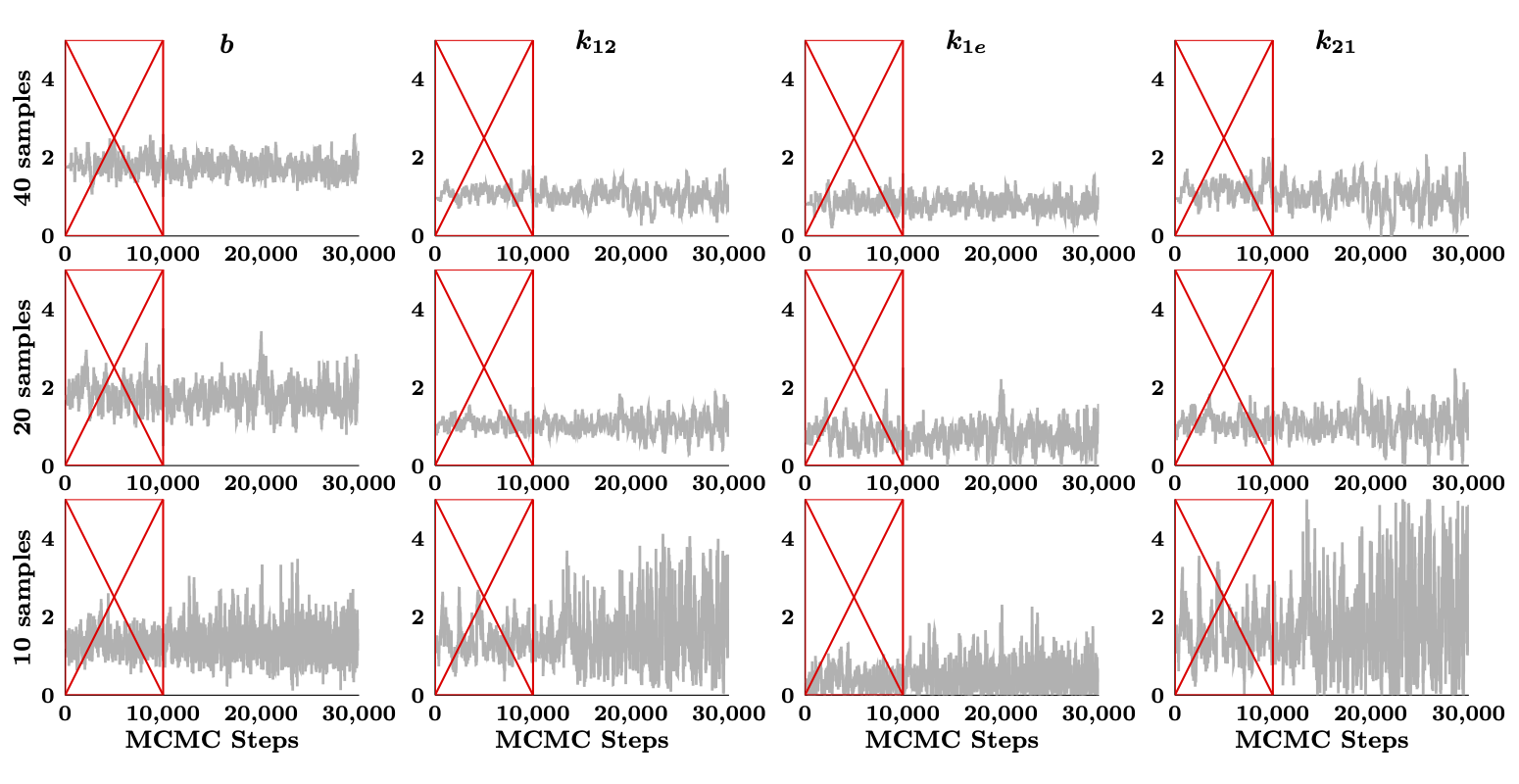

Supplement: S3 Fig — Each row corresponds to a sparsity level. The red boxes indicate samples discarded as burn-in. (TIF) [file pcbi.1010651.s003.tif]

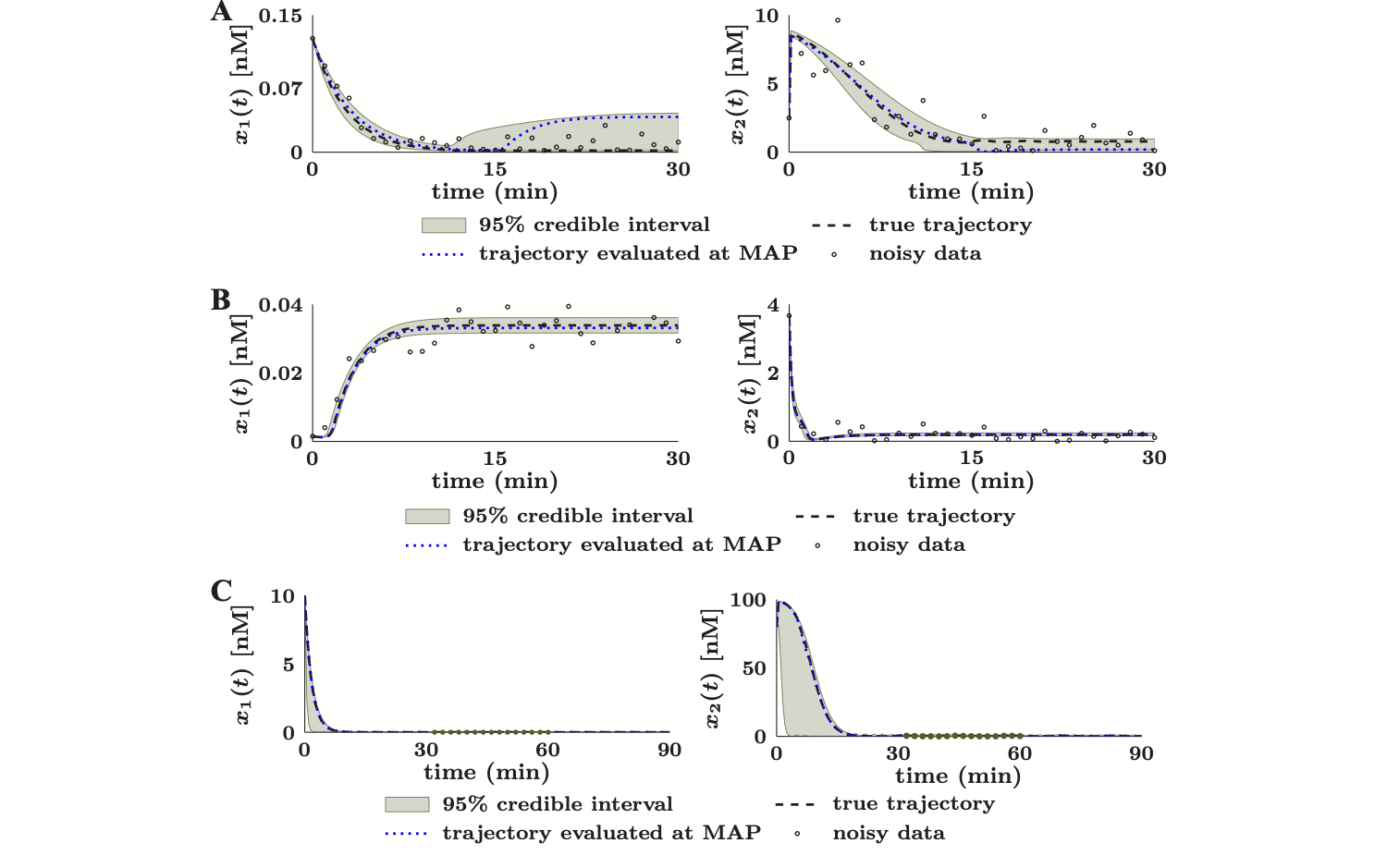

Supplement: S4 Fig — (A) Trajectories for the high steady-state; corresponds to Fig 5D. (B) Trajectories for the low steady-state; corresponds to Fig 5C. (C) Trajectories for limit cycle oscillations; corresponds to Fig 6B. (TIF) [file pcbi.1010651.s004.tif]

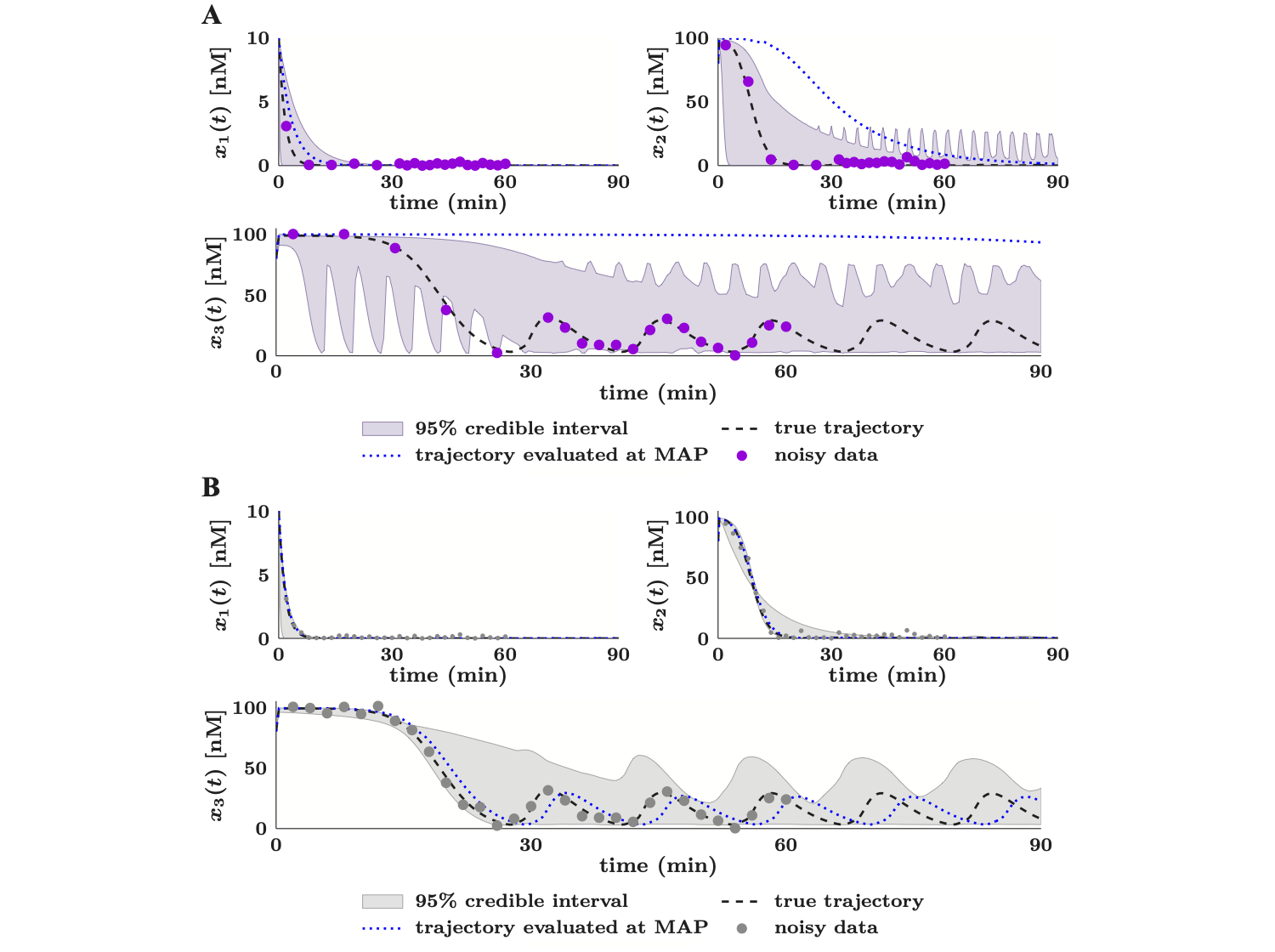

Supplement: S5 Fig — (A) Distributions for the data with equidistant sampling; corresponds to Fig 7C. (B) Distributions for the data with non-equidistant sampling; corresponds to Fig 7D. (TIF) [file pcbi.1010651.s005.tif]

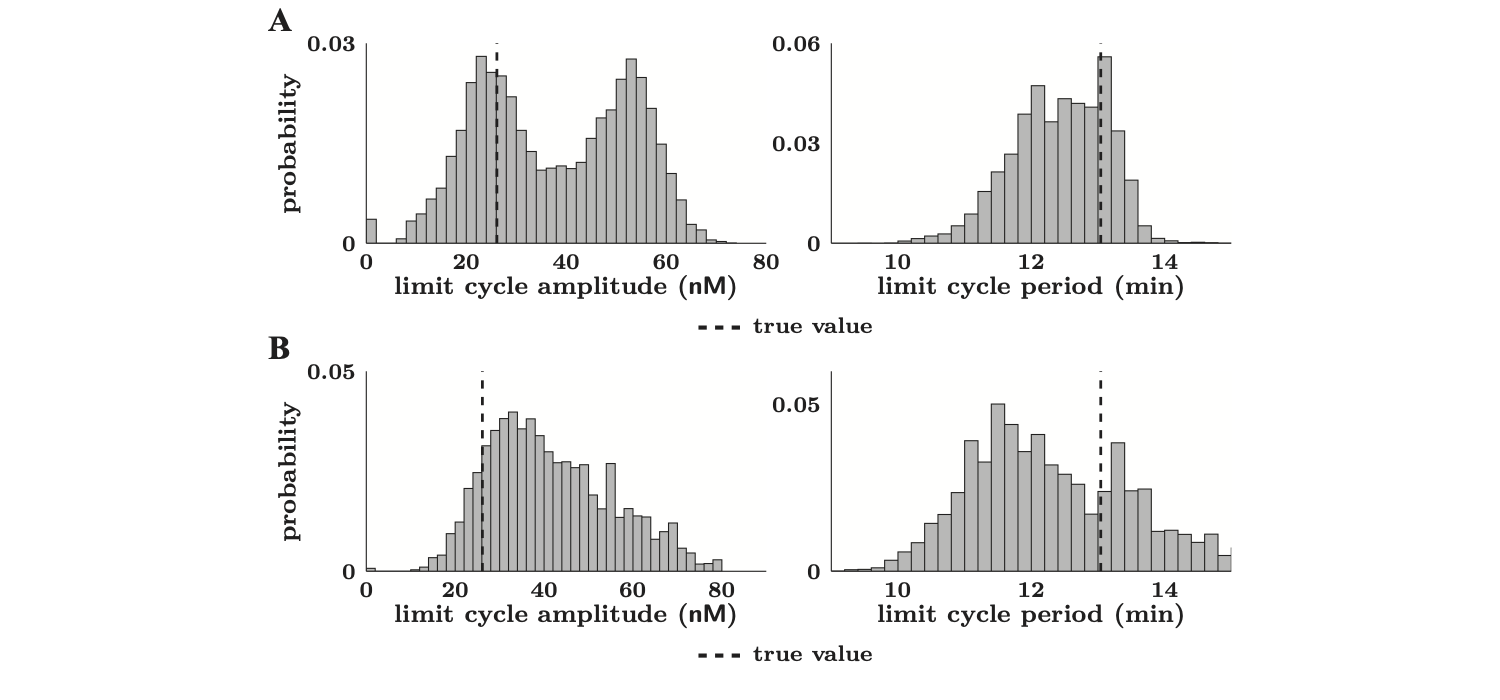

Supplement: S6 Fig — (A) Corresponds to S5 Fig. (B) Corresponds to Fig 7D and S5 Fig (TIF) [file pcbi.1010651.s006.tif]

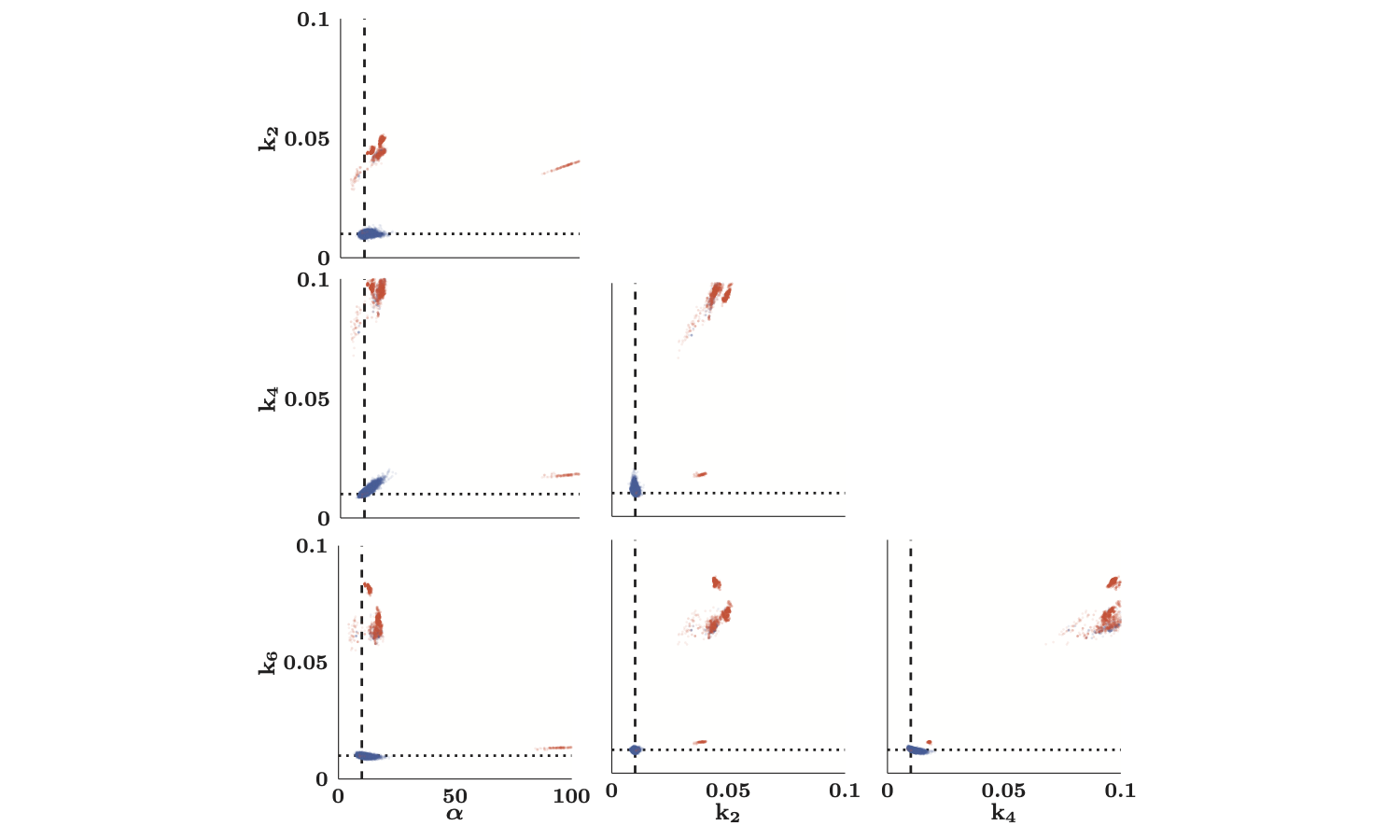

Supplement: S7 Fig — Each plot has 30,000 points that are colored according to the nature of the simulated dynamics with that parameter set. Simulations with blue points produce limit cycle oscillations, and those with red points produce fixed points. Darker regions indicate a higher probability of observing the corresponding parameter values. (TIF) [file pcbi.1010651.s007.tif]

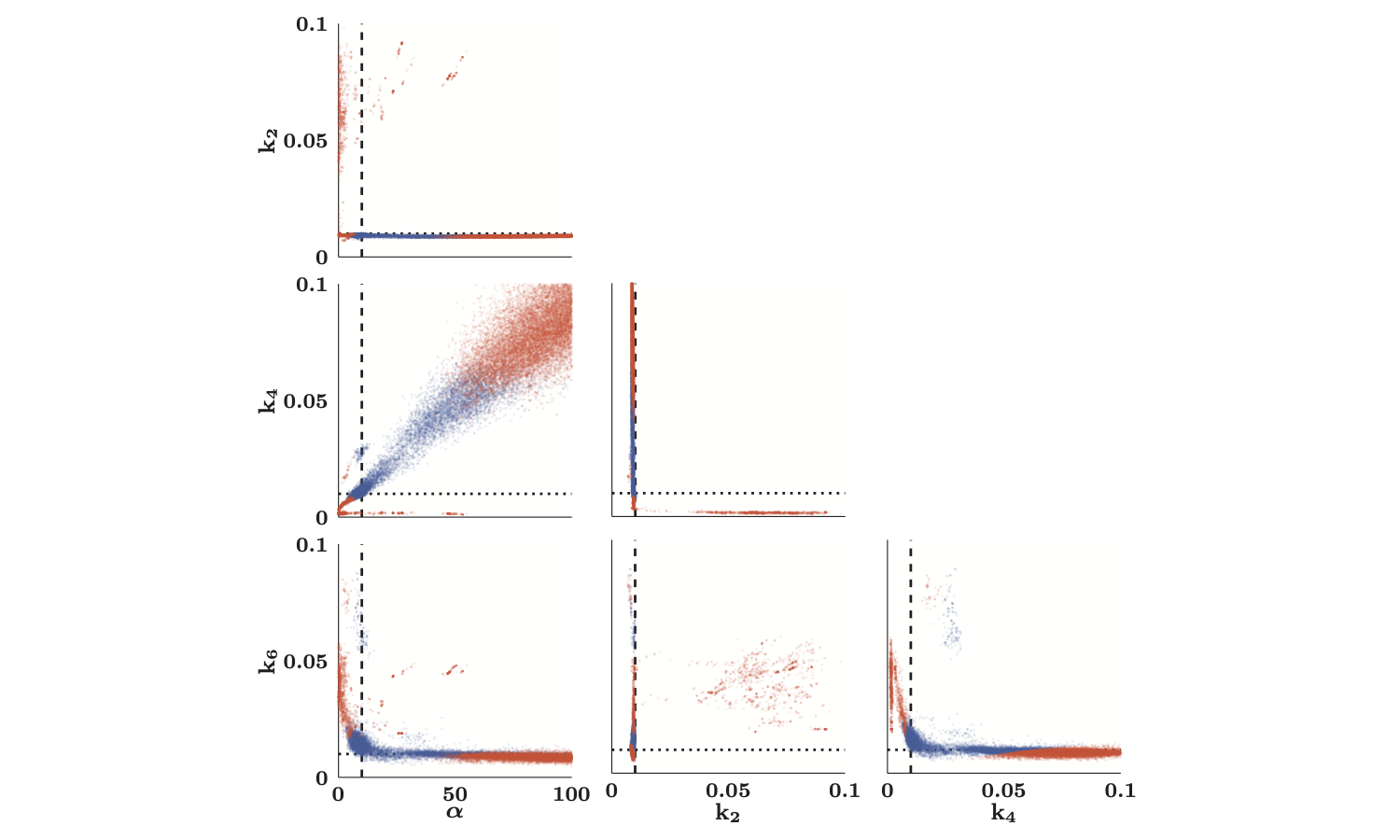

Supplement: S8 Fig — Each plot has 30,000 points that are colored according to the nature of the simulated dynamics with that parameter set. Simulations with blue points produce limit cycle oscillations, and those with red points produce fixed points. Darker regions indicate a higher probability of observing the corresponding parameter values. (TIF) [file pcbi.1010651.s008.tif]

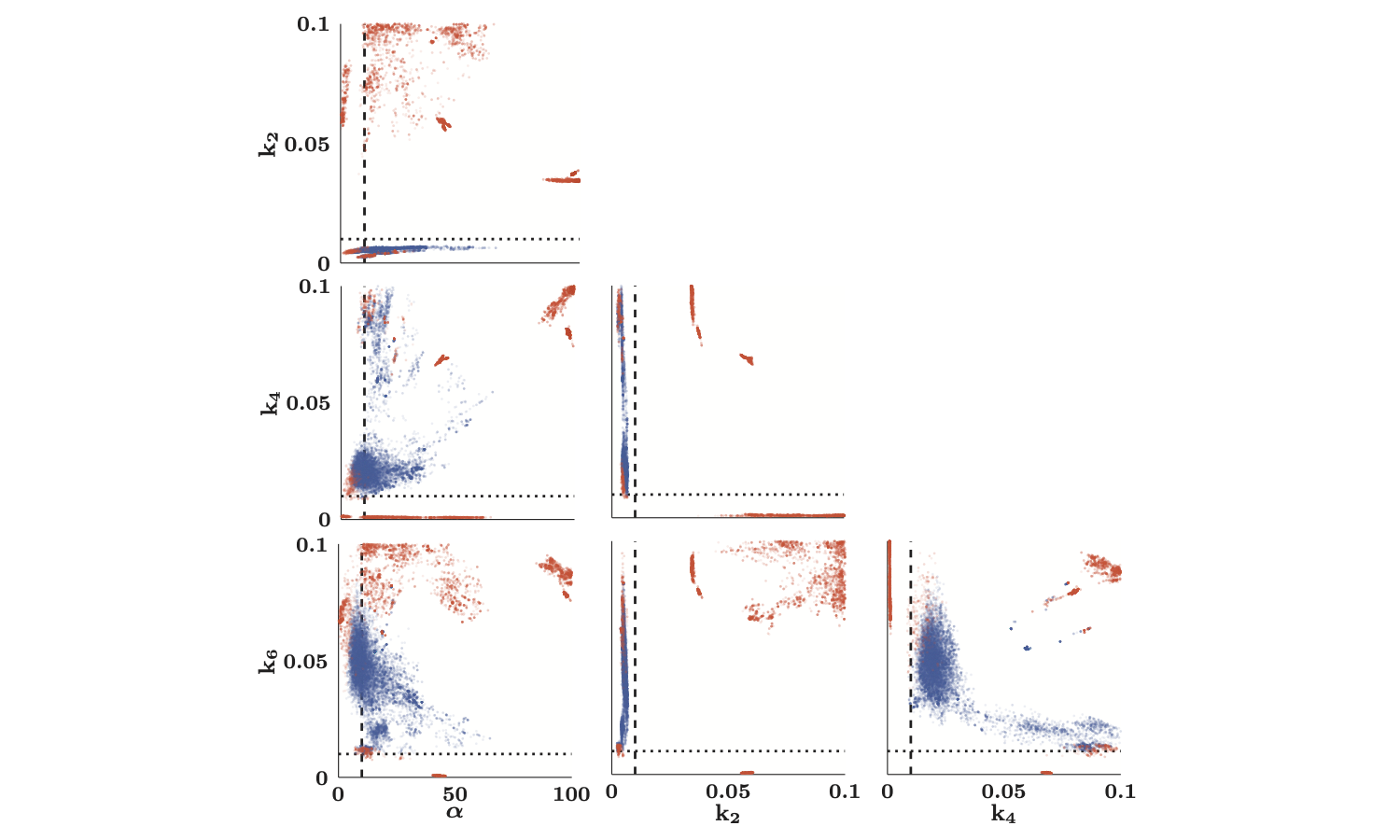

Supplement: S9 Fig — Each plot has 30,000 points that are colored according to the nature of the simulated dynamics with that parameter set. Simulations with blue points produce limit cycle oscillations, and those with red points produce fixed points. Darker regions indicate a higher probability of observing the corresponding parameter values. (TIF) [file pcbi.1010651.s009.tif]

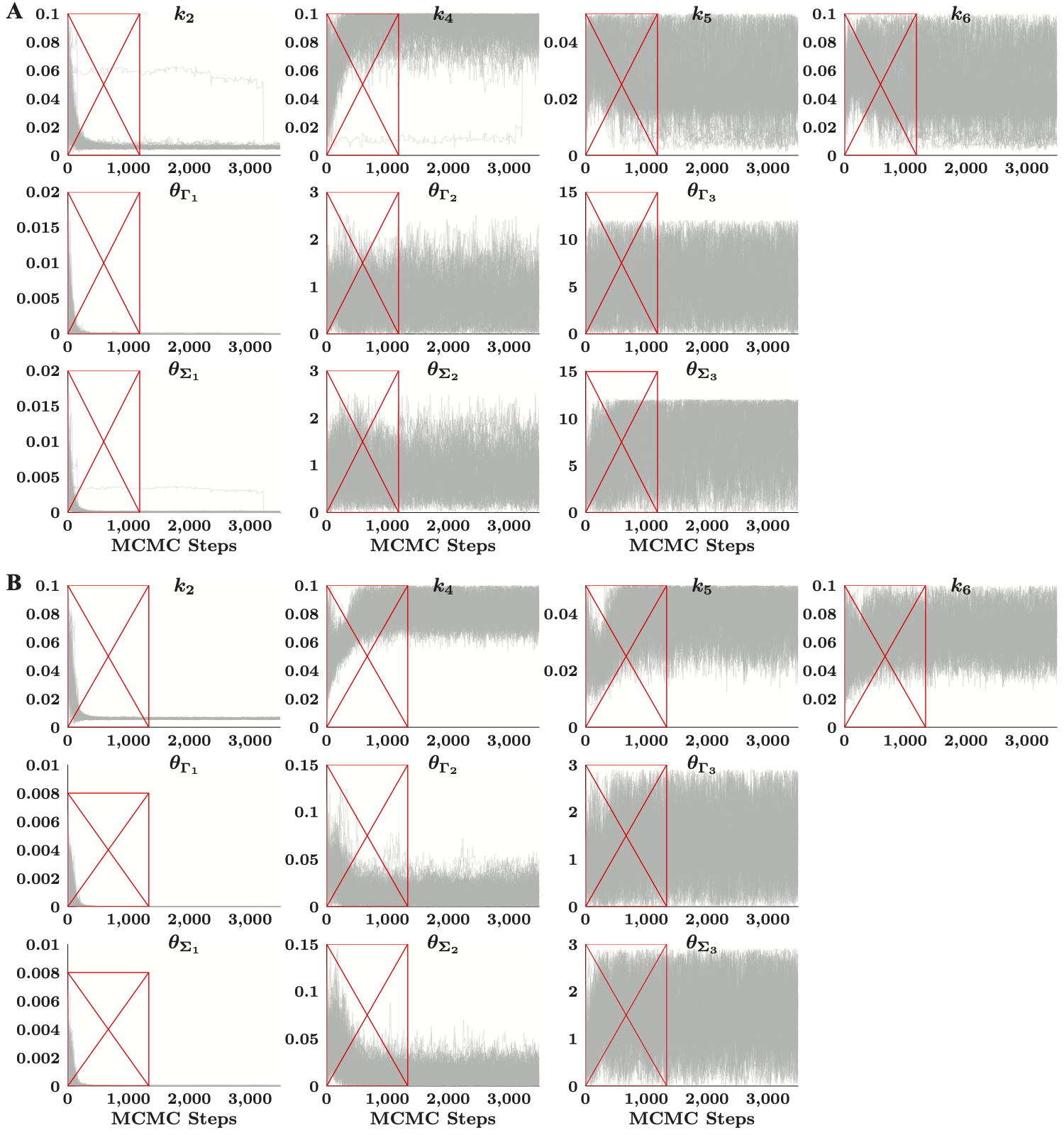

Supplement: S10 Fig — (A) Low steady-state, Fig 5A. (B) High steady-state, Fig 5B. The process noise covariances are θΓi and θΣi are the measurement noise covariances. The red boxes indicate samples discarded as burn-in. (TIF) [file pcbi.1010651.s010.tif]

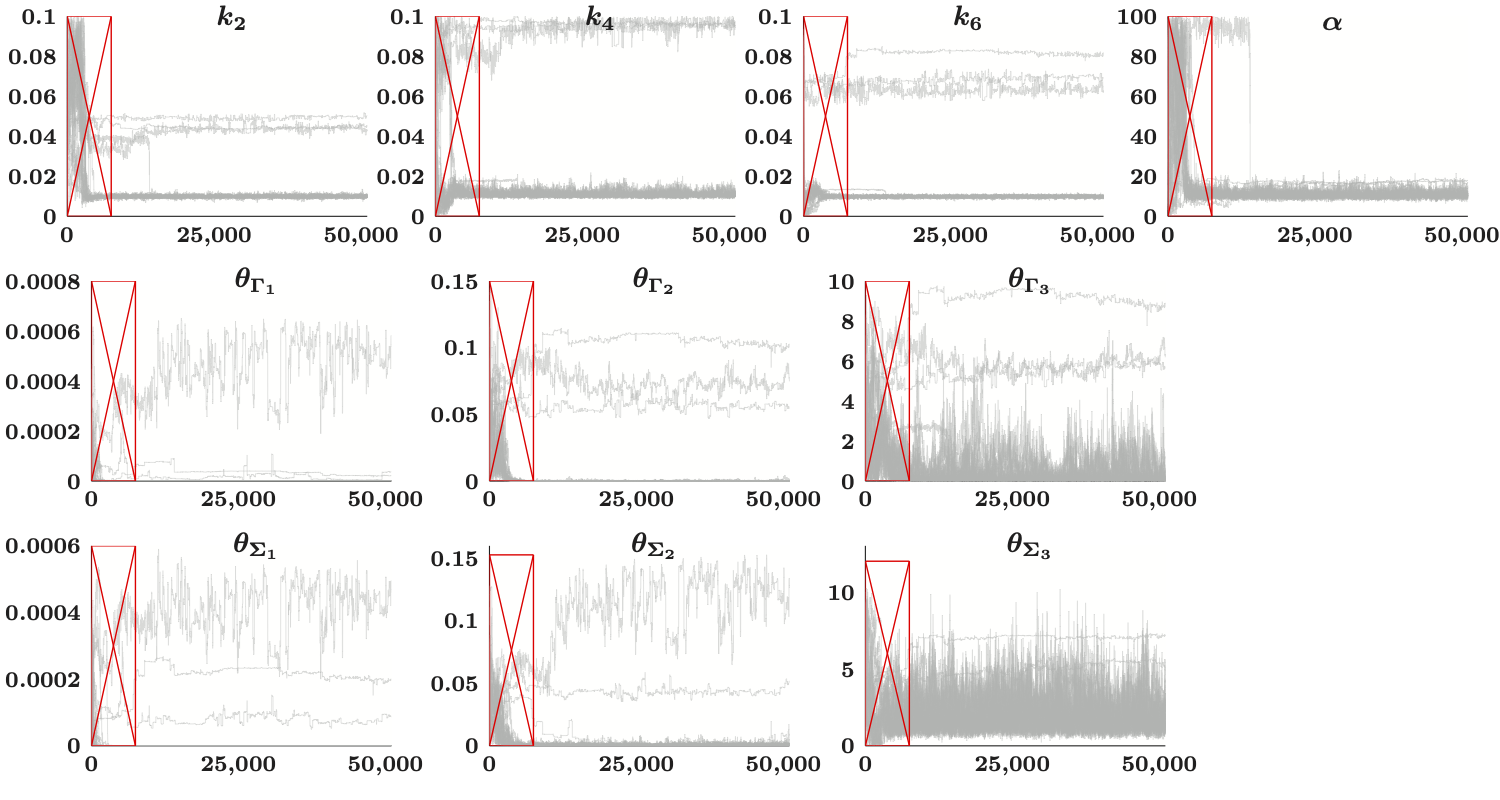

Supplement: S11 Fig — The process noise covariances are θΓi and θΣi are the measurement noise covariances. The red boxes indicate 7,477 samples per chain discarded as burn-in. (TIF) [file pcbi.1010651.s011.tif]

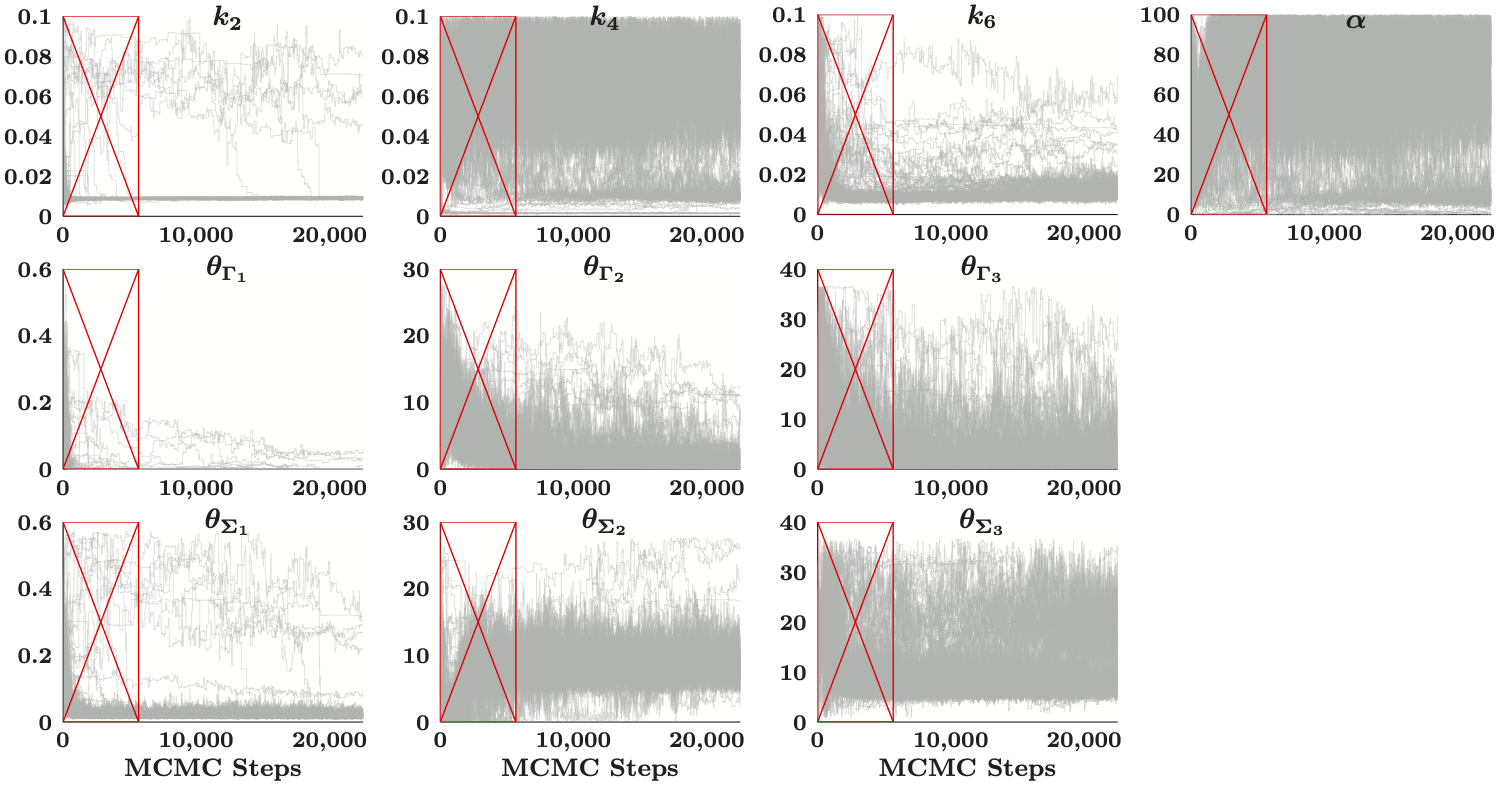

Supplement: S12 Fig — The process noise covariances are θΓi and θΣi are the measurement noise covariances. The red boxes indicate 5,669 samples per chain discarded as burn-in. (TIF) [file pcbi.1010651.s012.tif]

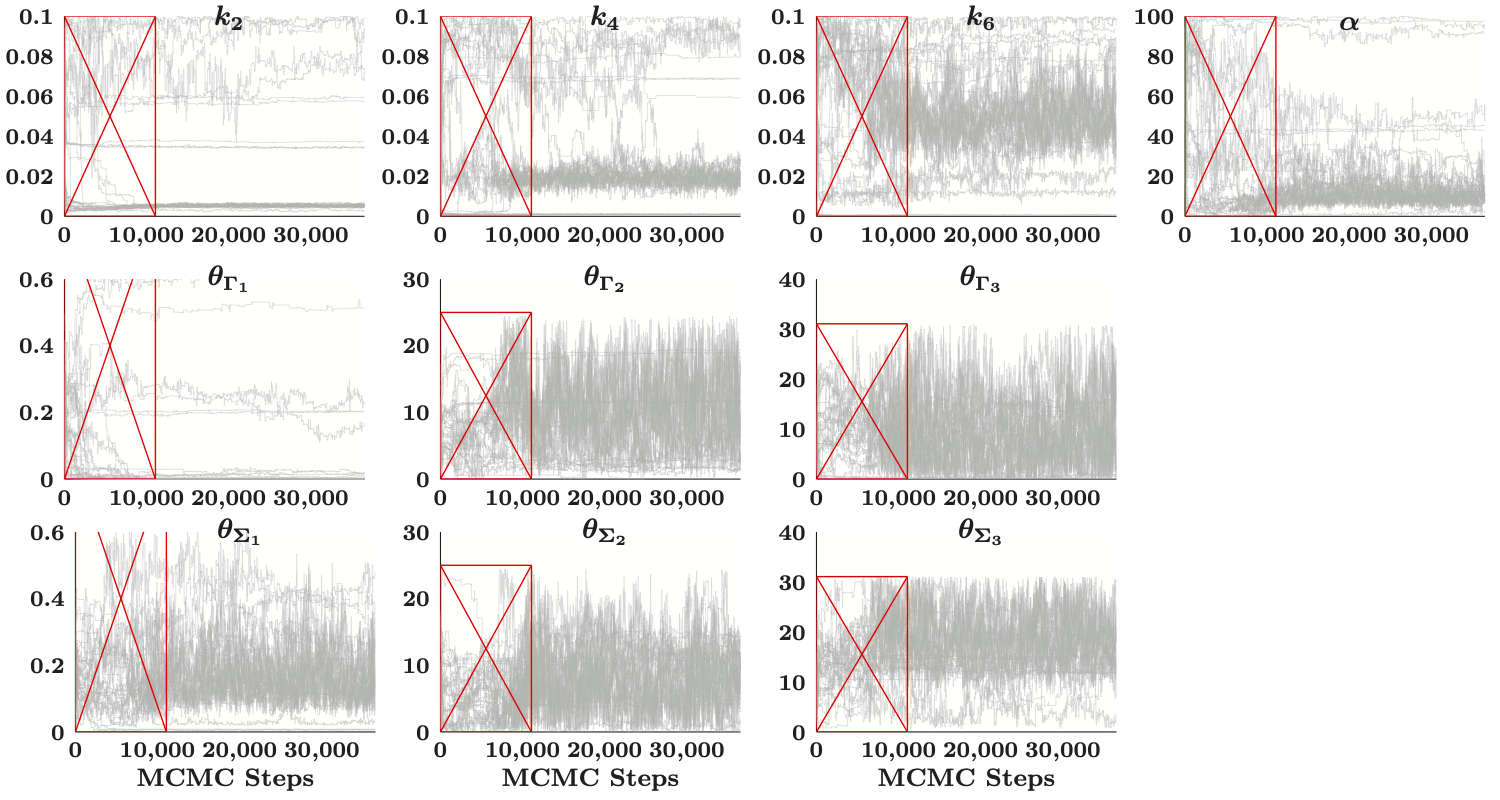

Supplement: S13 Fig — The process noise covariances are θΓi and θΣi are the measurement noise covariances. The red boxes indicate 11,065 samples per chain discarded as burn-in. (TIF) [file pcbi.1010651.s013.tif]

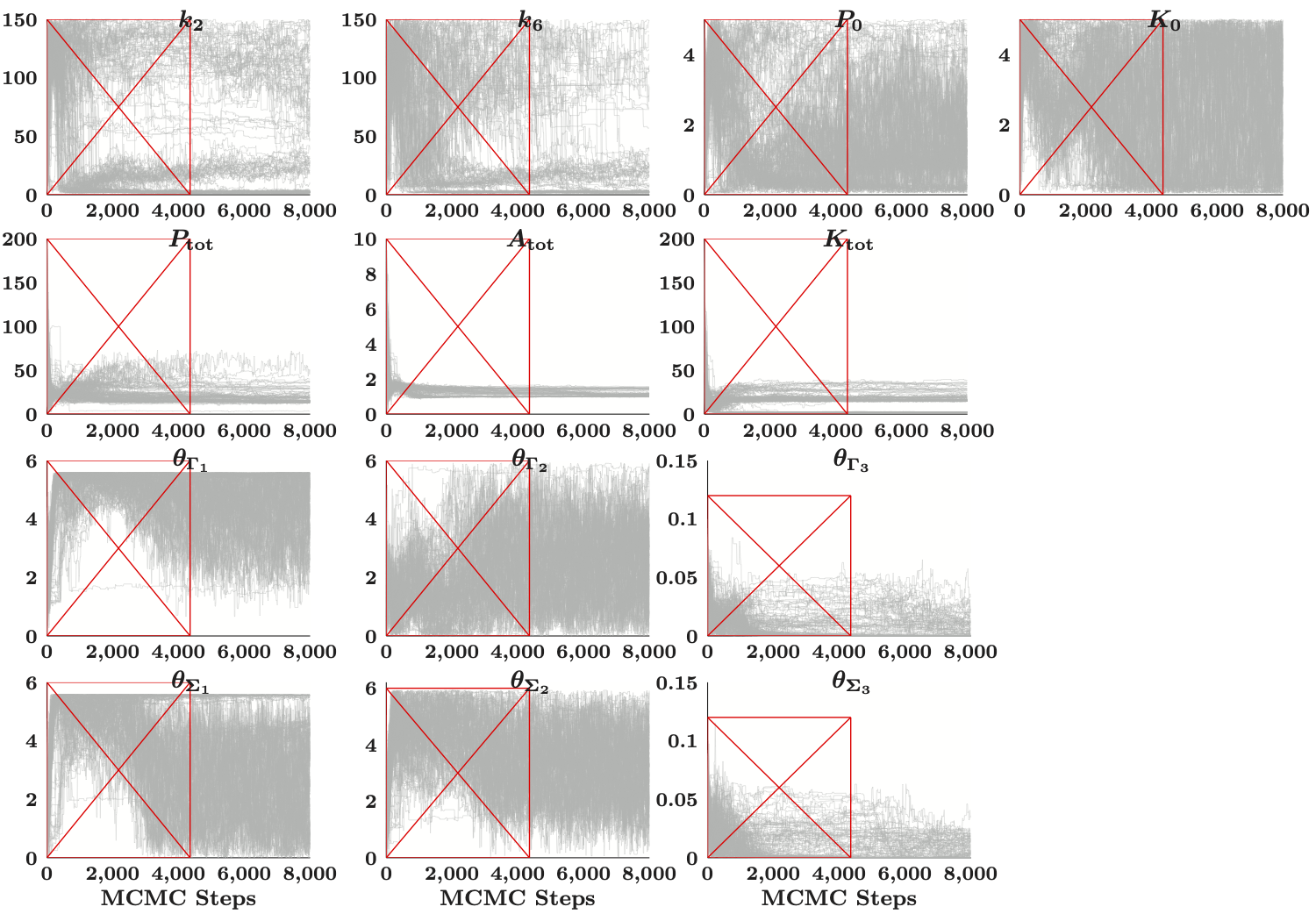

Supplement: S14 Fig — The red boxes indicate samples discarded as burn-in. (TIF) [file pcbi.1010651.s014.tif]
