# Supplementary material for: Bayesian parameter estimation for dynamical models in systems biology
Source: PLoS Comput Biol. 2022 Oct 21;18(10):e1010651. doi: 10.1371/journal.pcbi.1010651 (PMC9629650; doi:10.1371/journal.pcbi.1010651)
Supplement: S1 Text — Section 1. Structural identifiability and global sensitivity analyses are key to successful parameter estimation. Section 2. Runtime analysis of the CIUKF-based likelihood function. (PDF) [file pcbi.1010651.s015.pdf]

S1 Text: Bayesian parameter estimation for dynamical models in systems biology

Nathaniel J. Linden, Boris Kramer, Padmini Rangamani

1. Structural identifiability and global sensitivity analyses are key to successful parameter estimation

We propose an uncertainty quantification framework that applies structural identifiability and global sensitivity analysis to reduce the number of parameters to be estimated. This preprocessing is necessary as failure to reduce the parameter set to the identifiable and influential parameters led to a more difficult estimation problem and increased parameter uncertainty. To highlight these effects, we varied the preprocessing analysis that we used to reduce the set of free parameters for the MAPK model in the bistable regime. In particular, we tested two additional cases; one where we only used structural identifiability analysis to reduce the parameter space and another where we did not perform any preprocessing. Based on our previous experience, we predicted that parameter uncertainty would increase as we eliminated each parameter reduction preprocessing step.

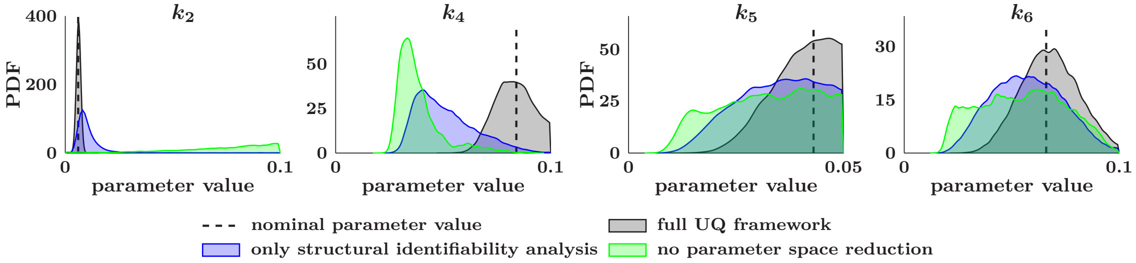

Figure 1: Marginal posterior distributions for k_2, k_4, k_5, and k_6 highlight the effects of excluding one or both of the structural identifiability and global sensitivity analyses. We visualize all distributions by fitting a kernel density estimator to MCMC samples. The grey-shaded distributions, labeled "full UQ framework," are the posterior distributions for the estimated parameters after applying the entire proposed UQ framework to reduce the free parameters to $\boldsymbol{\theta}_f = [k_2,\ k_4,\ k_5,\ k_6]^\top$. The blue-shaded distributions, labeled "only structural identifiability analysis," are the posterior distributions after using only structural identifiability analysis to reduce the parameter set to $\boldsymbol{\theta}_f^{\text{no GSA}} = [k_1,\ k_2,\ k_3\ k_4,\ k_5,\ k_6,\ \alpha]^\top$. The green-shaded distributions, labeled "no parameter space reduction," are the posterior distributions for the full set of free parameters $\boldsymbol{\theta}_f^{\text{no reduction}} = [k_1,\ k_2,\ k_3\ k_4,\ k_5,\ k_6,\ K_1,\ K_2,\ \alpha]^\top$. We only show the distributions for the four parameters that are common to all three analyses. In each case, we used CIUKF-MCMC with AIES to draw 3,500 MCMC samples with 150 walkers. We discarded 840, 1,342, and 1,485 samples per walker as burn-in for the full framework, structural identifiability only, and no reduction cases, respectively.

Based on the marginal parameter distributions in Figure 1, we conclude that structural identifiability and global sensitivity analysis are necessary for practical parameter estimation and uncertainty quantification. We estimated all parameters from the same data set generated from the MAPK low steady state in the bistable regime (results shown in Fig 5 in the main text). The posterior distributions of the reduced set of free model parameters $\boldsymbol{\theta}_f = [k_2,\ k_4,\ k_5,\ k_6]^\top$ that we found by applying the complete proposed framework (black line with grey shading) have peaks well-aligned with the nominal parameter values. Repeating the estimation with the set of free parameters $\boldsymbol{\theta}_f^{\text{no GSA}} = [k_1,\ k_2,\ k_3\ k_4,\ k_5,\ k_6,\ \alpha]^\top$ that we found by only using structural identifiability analysis for parameter reduction yields estimated posterior distributions (blue line and shading) with much greater uncertainties. Here, the peaks of the distributions for k_2 and k_4 do not align with the nominal values, and all four distributions are wider than in the previous case.

Lastly, estimation of the full set of free model parameters (no preprocessing to reduce the parameter set) $\boldsymbol{\theta}_f^{\text{no reduction}} = [k_1,\ k_2,\ k_3\ k_4,\ k_5,\ k_6,\ K_1,\ K_2,\ \alpha]^\top$ resulted in posterior distributions with even greater uncertainty which indicate lack of MCMC convergence. In this case, the posterior distributions place little probability on the nominal values of k_2 and k_4; thus, the predictions for these parameters would be incorrect. We used AIES for MCMC with 3,500 samples and 150 walkers for all three cases. These standard MCMC settings highlight how reducing the parameter space with structural identifiability and global sensitivity analyses lead to a better-posed estimation problem. Overall, each preprocessing step improves MCMC convergence and reduces uncertainty in the estimated parameters.

2. Runtime analysis of the CIUKF-based likelihood function

UKF-MCMC, and thus CIUKF-MCMC, poses an increased computational cost compared to estimation methods that do not consider all sources of uncertainty [1]. To better understand how this increased computational burden affects parameter estimation, we used the `timeit()` function in Matlab to time the execution of a CIUKF-based likelihood function and a Gaussian likelihood function Eq (7) in the main text that ignores model form uncertainty and that is representative of standard approaches [2, 3, 4, 5]. Specifically, we ran `timeit()` 20 times for each likelihood function evaluated for the MAPK model with the data from the low steady state, seen in Fig 5.A in the main text. We found that, on average, the CIUKF-based likelihood function takes 81 times longer (0.278 seconds) than the Gaussian likelihood (0.0034 seconds). All timing was performed on a laptop with a 2.6Ghz 6-core Intel Core i7 (Intel Corporation, Santa Clara, CA) processor with 32 GB of RAM.

To better understand what computations contribute to the observed runtimes of the CIUKF-based likelihood, we used the `profiler` in Matlab to analyze a single call to the likelihood function. We found that the CIUKF-based likelihood execution spent approximately 60% of the time on calls to `ode15s()`, which we used to discretize the ordinary differential equation model. Additionally, another 30% of the time was spent running `quadprog()` during the update step of the constrained interval unscented Kalman filter. Based on these findings, we conclude that developing better-optimized codes for these steps will likely accelerate the CIUKF-based likelihood function and, thus, shorten MCMC runtimes.

References

[1] Galioto N, Gorodetsky AA. Bayesian system ID: Optimal management of parameter, model, and measurement uncertainty. Nonlinear Dyn. 2020;102(1):241–267.

[2] Smith RC. Uncertainty quantification: Theory, implementation, and applications. vol. 12. SIAM; 2013.

[3] Mortlock RD, Georgia SK, Finley SD. Dynamic regulation of JAK-STAT signaling through the prolactin receptor predicted by computational modeling. Cell Mol Bioeng. 2021;14(1):15–30.

[4] Ghasemi O, Lindsey ML, Yang T, Nguyen N, Huang Y, Jin YF. Bayesian parameter estimation for nonlinear modelling of biological pathways. BMC Syst Biol. 2011;5 Suppl 3:S9.

[5] Wilkinson DJ. Bayesian methods in bioinformatics and computational systems biology. Brief Bioinform. 2007;8(2):109–116.
