# Supplementary material for: Bayesian parameter estimation for dynamical models in systems biology
Source: PLoS Comput Biol. 2022 Oct 21;18(10):e1010651. doi: 10.1371/journal.pcbi.1010651 (PMC9629650; doi:10.1371/journal.pcbi.1010651)
Supplement: S1 Table — All listed values have units of one over time. (PDF) [file pcbi.1010651.s016.pdf]

Parameter	Nominal Value	Range
k_{1e}	1	$[0,\ 5]$
k_{12}	1	$[0,\ 5]$
k_{21}	1	$[0,\ 5]$
b	2	$[0,\ 10]$
