# Supplementary material for: Bayesian parameter estimation for dynamical models in systems biology
Source: PLoS Comput Biol. 2022 Oct 21;18(10):e1010651. doi: 10.1371/journal.pcbi.1010651 (PMC9629650; doi:10.1371/journal.pcbi.1010651)
Supplement: S2 Table — Note: For the oscillatory dynamics, the range for k5 is [1 × 10^−5^, 0.05]. (PDF) [file pcbi.1010651.s017.pdf]

Parameter	Nominal Value: Limit-cycle	Nominal Value: Bistable	Range
S_{1t}	100 nM	0.22 nM	[0, 100]
S_{2t}	100 nM	10 nM	[0, 100]
S_{3t}	100 nM	53 nM	[0, 100]
k_1	0.1 nM $\cdot$ s^{-1}	0.0012 nM $\cdot$ s^{-1}	[0, 0.05]
k_2	0.01 nM $\cdot$ s^{-1}	0.006 nM $\cdot$ s^{-1}	[0, 0.1]
k_3	0.01 nM $\cdot$ s^{-1}	0.049 nM $\cdot$ s^{-1}	[0, 0.05]
k_4	0.01 nM $\cdot$ s^{-1}	0.084 nM $\cdot$ s^{-1}	[0, 0.1]
k_5	0.01 nM $\cdot$ s^{-1}	0.043 nM $\cdot$ s^{-1}	[0, 0.05]
k_6	0.01 nM $\cdot$ s^{-1}	0.066 nM $\cdot$ s^{-1}	[0, 0.1]
n_1	10	5	[5, 10]
K_1	1 nM	9.5 nM	[0, 10]
n_2	15	10	[5, 10]
K_2	8 nM	15 nM	[0, 20]
α	10	95	[0, 100]
