# Supplementary material for: Bayesian parameter estimation for dynamical models in systems biology
Source: PLoS Comput Biol. 2022 Oct 21;18(10):e1010651. doi: 10.1371/journal.pcbi.1010651 (PMC9629650; doi:10.1371/journal.pcbi.1010651)
Supplement: S3 Table — (PDF) [file pcbi.1010651.s018.pdf]

**State-variable**	**Limit cycle**	**Bistable: low steady state**	**Bistable: high steady state**
$x_1(0)$	10 nM	0.0015 nM	0.1245 nM
$x_2(0)$	80 nM	3.6678 nM	2.4870 nM
$x_3(0)$	80 nM	28.7307 nM	31.2623 nM
