# Supplementary material for: Bayesian parameter estimation for dynamical models in systems biology
Source: PLoS Comput Biol. 2022 Oct 21;18(10):e1010651. doi: 10.1371/journal.pcbi.1010651 (PMC9629650; doi:10.1371/journal.pcbi.1010651)
Supplement: S4 Table — The ranges are given by [0.1·θi*,10·θ*I], where θi* is the nominal value. Note: we do not include ranges for n1 and n2 because these parameters are always set to the nominal values. (PDF) [file pcbi.1010651.s019.pdf]

**Parameter**	**Nominal Value**	**Range**
k_1	2 s^{-1}	$[0.2,\ 20]$
k_2	15 s^{-1}	$[1.5,\ 150]$
k_3	1 s^{-1}	$[0.1,\ 100]$
k_4	120 s^{-1}	$[12,\ 1200]$
k_5	2 s^{-1}	$[0.2,\ 20]$
k_6	15 s^{-1}	$[1.5,\ 150]$
k_7	1 s^{-1}	$[0.1,\ 10]$
k_8	80 s^{-1}	$[8.0,\ 800]$
c_1	1	$[0.1,\ 10]$
c_2	1	$[0.1,\ 10]$
c_3	6 s^{-1}	$[0.6,\ 60]$
c_4	8 s^{-1}	$[0.8,\ 80]$
K_{m1}	10 μM	$[1.0,\ 100]$
K_{m2}	0.3 μM	$[0.03,\ 3]$
K_{m3}	4 μM	$[0.4,\ 40]$
K_{m4}	10 μM	$[1.0,\ 100]$
K_{m5}	1 μM	$[0.1,\ 10]$
K_0	0.5 μM	$[0.05,\ 5]$
P_0	0.5 μM	$[0.05,\ 5]$
K_{tot}	20 μM	$[2,\ 200]$
P_{tot}	20 μM	$[2,\ 200]$
A_{tot}	1	$[0.1,\ 10]$
n_1	4	–
n_2	3	–
